# The ADVANCE toolkit: Automated descriptive video annotation in naturalistic child environments

**DOI:** 10.3758/s13428-025-02883-0

**Published:** 2025-11-19

**Authors:** Naomi K. Middelmann, Jean-Paul Calbimonte, Emily B. Wake, Manon E. Jaquerod, Nastia Junod, Jennifer Glaus, Olga Sidiropoulou, Kerstin J. Plessen, Micah M. Murray, Matthew J. Vowels

**Affiliations:** 1 Division of Child and Adolescent Psychiatry, Department of Psychiatry, Lausanne University Hospital Center and University of Lausanne, Lausanne, Switzerland; 2 The Sense Innovation and Research Center, Lausanne and Sion, Switzerland; 3 Institute of Informatics, University of Applied Sciences and Arts Western Switzerland (HES-SO Valais), Sierre, Switzerland; 4 The Institute of Psychology, University of Lausanne, Lausanne, Switzerland; 5 The Radiology Department, Lausanne University Hospital Center and University of Lausanne, Lausanne, Switzerland; 6 The Centre for Vision, Speech and Signal Processing, University of Surrey, Guildford, UK

**Keywords:** Video annotation, Pose estimation, Computer vision, Object tracking, Motion tracking

## Abstract

Video recordings are commonplace for observing human and animal behaviours, including interindividual interactions. In studies of humans, analyses for clinical applications remain particularly cumbersome, requiring human-based annotation that is time-consuming, bias-prone, and cost-ineffective. Attempts to use machine learning to address these limitations still oftentimes require highly standardised environments, scripted scenarios, and forward-facing individuals. Here, we provide the ADVANCE toolkit, an automated video annotation pipeline. The versatility of ADVANCE is demonstrated with schoolchildren and adults in an unscripted clinical setting within an art classroom environment that included 2–5 individuals, dynamic occlusions, and large variations in actions. We accurately detected each individual, tracked them simultaneously throughout the duration of the recording (including when an individual left and re-entered the field of view), estimated the position of their skeletal joints, and labelled their poses. By resolving challenges of manual annotation, we radically enhance the ability to extract information from video recordings across different scenarios and settings. This toolkit reduces clinical workload and enhances the ethological validity of video-based assessments, offering scalable solutions for behaviour analyses in naturalistic contexts.

## Introduction

Video recordings pervade the study of human and animal behaviour (Bednarski et al., 2022; Lauer et al., 2022), as they arguably provide a reliable record of events that can be viewed and reviewed effectively at one’s leisure. In clinical research, in particular, there are wide-ranging challenges to video recordings and their annotation, which are further exacerbated when studying children. In terms of the recording contexts, the scenarios are typically scripted or semi-structured, in an attempt to isolate and assess specific (dys)functions. The position of the participant(s) is oftentimes restricted, such as to be front-facing. Sensors or other external devices are often worn to track movements or similar gestures (Muñoz-Organero et al., 2018; Wood et al., 2009). Such unconventional and unnatural contexts can skew the perception (and recording) of the actual behaviours of interest (FitzGibbon et al., 2024). This can be further compounded by an audience effect (Hamilton & Lind, 2016), by biases of the rapport between the participant and the examiners involved in the interviews (Moffett et al., 2020; Sonuga-Barke et al., 2013), by the cultural backgrounds of the participants (Quintela Do Carmo et al., 2024), the linguistic abilities of the participants and whether or not they are using their native language (Cormier et al., 2022; Fernald et al., 2013), as well as by the added stress of being observed/filmed by an often unknown experimenter or clinician (Quintela Do Carmo et al., 2024). Assumptions regarding the real-world and everyday relevance of semi-structured interviews therefore cannot be overlooked and moreover raise questions regarding whether such tools ultimately provide reliable assessments of human behaviour, clinical status (Gualtieri & Johnson, 2005; Reinecke et al., 1999), or the effectiveness of intervention approaches (Sempere-Tortosa et al., 2020).

Observational techniques applied to children must therefore account for these difficulties and complexities (Minder et al., 2018) and must operate within their natural settings, as a complement to the semi-structured settings, in order to quantitatively assess everyday behaviours (FitzGibbon et al., 2024; Posserud et al., 2014). These considerations similarly cascade to annotation techniques. Currently, scoring of videos remains overwhelmingly manual (Molloy et al., 2011). Doing so successfully necessitates both a sufficient number of trained individuals to conduct the recordings and interviews and maintenance of the annotators’ expertise. By extension, manual annotation is cumbersome and costly both financially and in terms of time-efficiency (Bulling et al., 2023). Progress has been made to implement video annotation techniques to recognize features and gestures related to clinical symptoms (Kojovic et al., 2021; Watson et al., 2024). By way of example, Lee et al. (Lee et al., 2023) filmed children playing a series of projected games in a specific lab setting in order to classify children with and without attention-deficit/hyperactivity disorder (ADHD). One limitation of their approach was that the child was filmed alone and without occlusion. Similarly, the path and actions were scripted and demonstrated by a robot that the children were instructed to imitate. Analytically, their approach capitalized upon the use of multiple cameras to surmount skeleton tracking and used coloured jackets to ensure accurate identification of individuals. Despite these advances, the methods have currently been demonstrated with individuals performing scripted movements and rely on the multiple sensors of the recording cameras (notably RGB-D). Such situations may not be applicable to standard recording scenarios nor to naturalistic behaviours of multiple individuals, including but not limited to when an individual leaves the field of view of the camera(s) and then later re-enters the scene, which results in a ‘re-identification’ challenge in computer vision. More generally, because such methods are specifically targeting diagnoses, they often put far less emphasis on detailing the characteristics of each individual’s movements or on how these may change with time, or under naturalistic or inter-individual contexts, or as a function of therapeutic interventions.

Current approaches to person tracking and re-identification in video analysis face significant limitations when applied to naturalistic clinical or behavioural contexts. Popular open-source tools like StrongSORT (Du et al., 2023), while effective in short-term tracking, generally overestimate the number of unique individuals due to their sensitivity to sustained occlusions and inability to limit the maximum number of identities. Alternative methods, such as DeepLabCut (Lauer et al., 2022; Mathis et al., 2018), require extensive manual annotation of video frames, making them labour-intensive and unsuitable for scalable or automated solutions. These shortcomings are particularly problematic in clinical applications, where precise and efficient tracking of freely moving individuals is essential for understanding complex behaviours without introducing artificial constraints or biases. Furthermore, such applications are constrained in terms of the maximum number of individuals for a given session, and so any methodological solution should benefit from, rather than be hampered by, this constraint. Surmounting these challenges requires methods that can reliably track and re-identify individuals across occlusions, operate without the need for large, annotated datasets, and maintain accuracy while minimizing the over-detection of identities.

In light of such considerations, automated annotation methods are needed that can operate in contexts involving multiple, freely moving individuals who are unrestricted and who are unencumbered by wearable devices; contexts we refer to for simplicity as ‘naturalistic’ insofar as the behaviours of the individuals are unconstrained and the art classroom has not been especially structured or equipped/uncluttered for the recordings. Here, we introduce ‘ADVANCE’, an automated pipeline to track and re-identify individuals following periods of occlusion (e.g., when a person is occluded by another person or object), overcoming certain limitations associated with alternative open-source methods. We also demonstrate the utility of these methods for downstream analyses, by classifying the pose of the individual as sitting or standing. It also provides a number of key advantages over existing alternatives which, for any given video, either tend to overestimate the number of unique individuals, or require a significant set of human-labelled examples for each of the individuals. The approach thereby reduces clinical workload and enhances ethological validity, offering scalable solutions for behaviour analysis in naturalistic contexts.

### Methods

#### Video dataset description

**Fig. 1:**
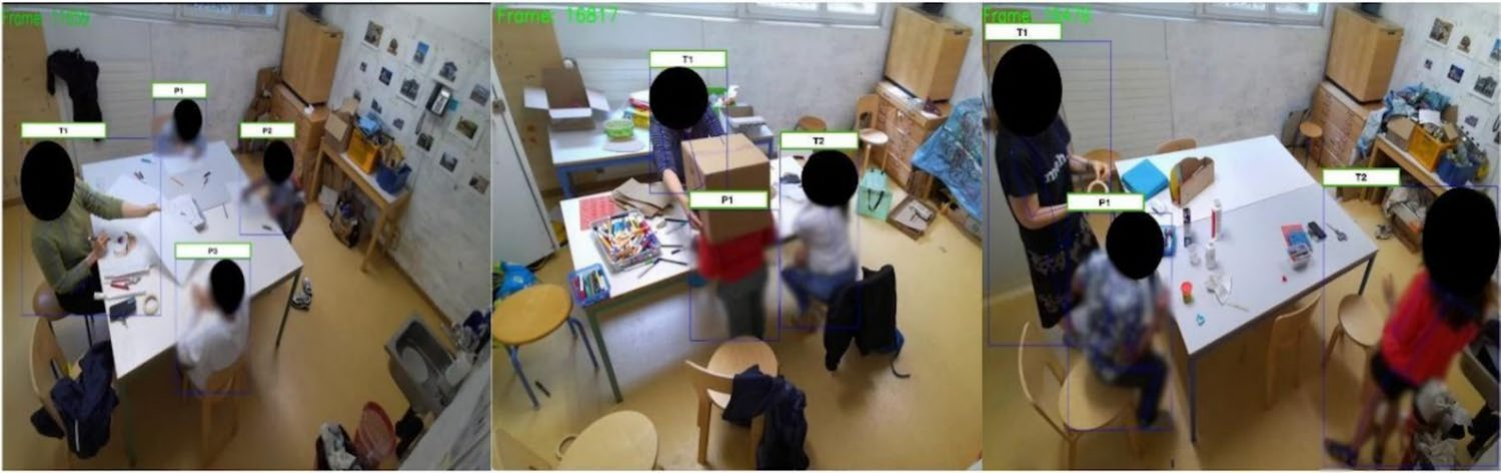
Example RGB video frames show the setting in which recordings were acquired and highlight the complexity of the scene and human behaviour. Videos included multiple individuals in varied positions/orientations with respect to the camera, instances of leaving and re-entering the field of view, and complex interactions with objects (e.g., wearing a cardboard box as in the middle panel). Videos have been blurred and blotted in the figure to protect identities

We leveraged a dataset of over 150 videos filmed during art therapy sessions led by a federally licensed art therapist at a day school centre under the auspices of the Psychiatry Department of the Lausanne University Hospital Center (CHUV). The videos were recorded in the context of a broader clinical research project and after obtaining written informed consent from the children’s parents or legal guardians. All recording procedures were approved by the cantonal ethics committee on research on humans (CER-VD protocol number 2022-01488) and conformed to the Declaration of Helsinki. Videos were captured using a Microsoft Azure Kinect camera mounted in the top corner of the art studio, offering an overview of the scene (Fig. 1). These weekly recordings span 20–24 weeks and collectively involved 14 children aged 7–12 at the time of recordings (fuller details are provided below). These children had received diagnoses of ADHD, ASD, learning and behavioural disabilities using standardised diagnostic questionnaires and clinical observations by licensed psychologists and psychiatrists of The Division of Child and Adolescent Psychiatry within The Department of Psychiatry at the Lausanne University Hospital Center and University of Lausanne.

In total, 36 video sessions were analysed for the purposes of the present study. Twelve of these contained two individuals, eight contained three individuals, eight contained four individuals, and eight contained five individuals. Among these videos were 15 unique children (all boys; aged 7–12 years; seven White and eight other) and six unique adults (all female; aged 25–50 years; five White and one other). Aside from the child/children present in the art studio, there were also 1–2 adults, at least one of whom was a federally licensed art therapist. Figure 1 displays examples of the complex scenes that were considered. The art studio setting is equipped with several tables, chairs, and objects. The children participated in individual or small-group art therapy sessions as part of their prescribed standard care. A sample processed video of adults in the studio, as well as estimated skeletons and the associated ID mapping, is accessible online (https://osf.io/xrsnw/files/osfstorage?view_only=9f09d98040d24616bf1c30af18ff31a3), which demonstrates the validity of the methods developed in this study.

Within this setting, the children could interact with different materials (e.g., pencils, pens, paints, clay, sand, etc.), construct with recycled materials, play with traditional toys (figurines or Lego), engage in symbolic play, make simple breads or baked goods, or interact with musical instruments. Sessions contained either semi-structured activities proposed by the therapist or were child-led. Children arrived in the studio, stood or sat at a large table in the middle of the room, and worked on an activity for approximately 30–45 min. Depending on the type of activities during a session, the child moved to different extents around the studio to retrieve materials from the cupboards or from different shelving units and work on one or several types of projects. Sessions were either composed of one adult with one child or 1–2 adults with 2–3 children. It is noteworthy that all adults were also active participants in the activities, either creating themselves or helping the children with the various materials. Activities performed by children and adults alike in these videos included: drawing and painting, Lego constructions, mask-making, sculpting with clay, playing with musical instruments, symbolic play, etc. These sessions included a wide range of activities as well as a high number of potential occlusions from both objects and other individuals.

#### Computational considerations

We tested the approach on a machine running Ubuntu 22.04 LTS, with an NVIDIA 2080ti, 126 GB of RAM, and an Intel i9-9900K 3.6 GHz CPU. The running times for different stages of the system were tested on a 42-s video consisting of 1,282 frames at 30 fps, with a resolution of 568 × 320, featuring two unique individuals. The results show that without skeleton pose estimation, the re-identification process of ADVANCE operates at 1.7 times real-time. In contrast, StrongSORT (also using YOLOv8 for the person detection) processes a video with two unique individuals on the same hardware at 0.8 times real-time. When OpenPose (Cao et al., 2018) is used for skeleton pose estimation, the processing time of ADVANCE is 6.9 times real-time. This processing time increases proportionately with the number of individuals.

#### Toolkit overview

**Fig. 2:**
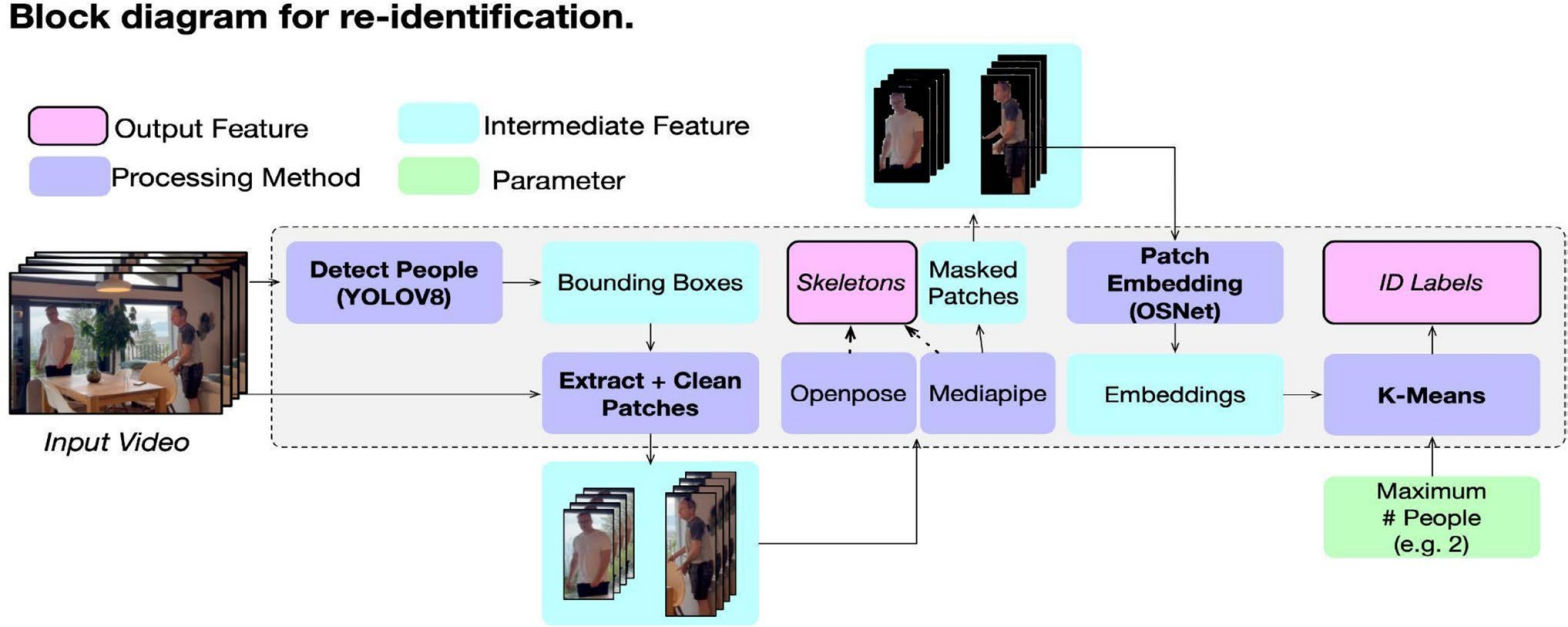
The pipeline for re-identifying individuals in a video

In contrast with existing techniques, the ADVANCE toolkit requires only the specification of the maximal number of unique identities within a video, thereby circumventing over-estimation of the number of individuals and the need for labelled examples. Figure 2 depicts the top-level diagram for the pipeline for person tracking and skeleton estimation. Here, we provide a synopsis. Code for the re-identification pipeline is available at https://osf.io/4mfsk/.

YOLOv8 (Varghese & Sambath, 2024) (Ultralytics implementation; https://github.com/ultralytics/ultralytics) is first used to detect the presence of individuals on a frame-by-frame basis, and to extract bounding box locations for each of the individuals (we use the yolov8x.pt model, available through the Ultralytics repository). At this stage, no tracking has been undertaken. The bounding boxes are used to extract image patches for the regions of the image containing detected individuals. The background in these image patches is then masked using Mediapipe (Lugaresi et al., 2019). Masking is important for the subsequent clustering, to prevent any changes in the background (e.g., furniture, wall colour, etc.) affecting the clustering of the image patch, which should instead be based solely on the appearance of the individual, rather than their environment.

It is at this stage that the skeleton positions for the individuals in the masked image patches are estimated using OpenPose (Cao et al., 2018). We would note, however, that these skeletons are not used for tracking. The masked patches are then embedded using OSNet from the torchreid library (Zhou & Xiang, 2019). OSNet is a model that has been pre-trained on re-identification tasks, and we therefore expect it to embed features that are discriminative with respect to the appearance of individuals. Indeed, we also tried generic image classification networks, including ResNet (He et al., 2016) variations, without as much success. The pretrained version used is the ‘osnet ain x1.0 msmt17’ model, available through the torchreid repository (Zhou & Xiang, 2019).

We then use the scikit-learn implementation of the k-means algorithm (Pedregosa et al., 2011) to cluster these image patch embeddings using the default hyperparameters (k-means++ initialization; automatic determination of the number of times the algorithm is run with different centroid seeds; a tolerance of 0.0001; 300 maximum iterations). It is the k-means algorithm which takes the maximum number of unique individuals as a parameter for the number of clusters. Following the assignment, we undertake some basic checks to ensure that the assignment of clusters is unique for each frame (i.e., two people for the same frame cannot be assigned the same identity, and if they are, the identification/cluster assignments are marked as mistaken).

Finally, if semantically meaningful labels are required, the researcher can also provide a mapping from the numeric cluster labels (e.g., 0, 1) to labels such as ‘Therapist’ and ‘Patient’. Note that, throughout this process, researchers must provide at least one key piece of information—i.e., the number of unique individuals in the video—and optionally a second, which is the mapping from cluster labels to semantically meaningful labels.

Once re-identification has been undertaken following the process outlined above, the researcher is free to use the extracted skeletons for a range of downstream tasks. We exemplify how they can be used for pose classification (sitting versus standing), but there are a wide range of possibilities at this stage. The most important thing is that the image patches, bounding boxes, and extracted features (such as skeletons) can be assigned consistently to the correct individual throughout the video, thereby enabling individual-specific processing.

## Results

### Previous approaches and their limitations

As a benchmark, we first used a well-established open-source tool for tracking (StrongSORT (Du et al., 2023)). StrongSORT is a popular multi-object tracking approach that incorporates both appearance similarity and motion to track. However, despite its general efficacy across benchmark tracking problems, we found that this option did not provide a ready means to hard-limit the number of detected people. More problematic for our use-case of videos of people coming in and out of the camera’s field of view, StrongSORT is sensitive to sustained occlusions and thus has difficulties re-identifying an individual who leaves and re-enters the field of view of the camera. StrongSORT resulted in successful short-term identification (i.e., consistent tracking during periods without substantial occlusion), but consistently over-estimated the number of identities. After periods of sustained occlusion, StrongSORT would assign brand new identities to previously tracked individuals, resulting in an over-estimation of the number of unique individuals in a video. This phenomenon is summarized in Table 1. At worst, 10 ‘extra’ individuals are detected than were actually present (*N* = 4). At best, 4 ‘extra’ individuals were detected (*N* = 2). An alternative, DeepLabCut (Lauer et al., 2022; Mathis et al., 2018), was not a viable option, principally because it requires manually annotating ~50–200 frames per video and is thus contrary to our objective of automation and scaling. Finally, *psifx* (Rochette & Vowels, 2024), an open-source feature extraction toolkit intended for human behavioural analysis, does not include multi-person tracking or pose classification options.

**Table 1:** The column Ground Truth (N) indicates the veridical number of individuals in a video, whereas the column StrongSORT Result indicates how many unique individuals were detected by StrongSORT (Du et al., 2023). There is consistent ‘over-detection’ of the number of unique individuals when running the StrongSORT re-identification approach. In contrast, our method has no difficulties, because the number of individuals is the sole requisite parameter specified in advance

Video	Ground Truth (N)	StrongSORT Result	Difference
A	2	6	4
B	2	7	5
C	3	8	5
D	3	9	6
E	4	10	6
F	4	14	10
G	5	11	6

### Accurate re-identification of multiple individuals

**Fig. 3:**
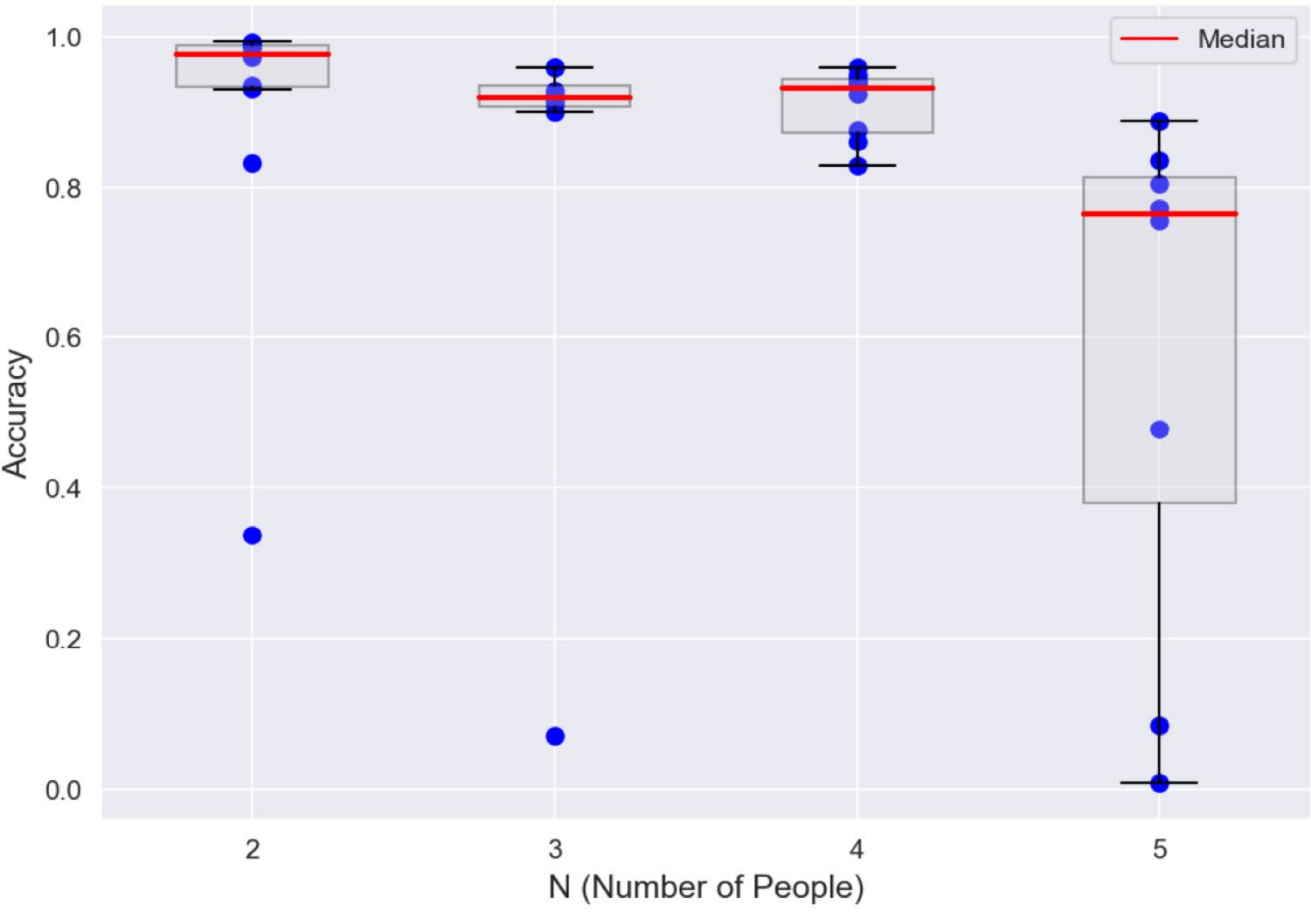
Re-identification accuracies for each of the 36 test videos (blue points) as a function of the different total number of unique individuals (N) that each video contained. The dataset includes 12 videos with 2 individuals, 8 videos with 3 individuals, 8 videos with 4 individuals, and 8 videos with 5 individuals. Box and whisker plots show the median (red line), interquartile range (IQR, box), and largest or smallest values within 1.5 times the IQR (upper and lower whiskers, respectively)

**Fig. 4:**
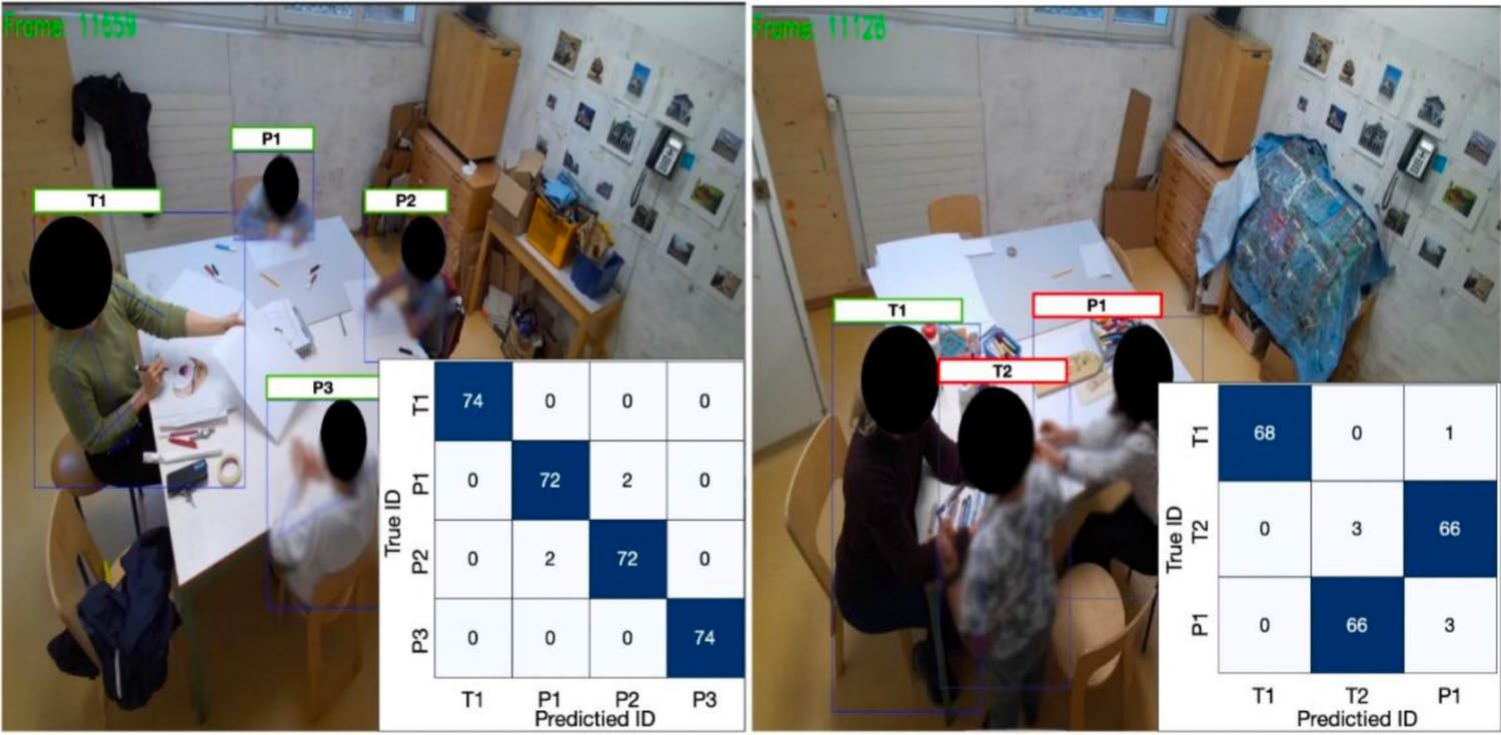
Success (**left**) and failure (**right**) cases for the re-identification, including per-individual confusion matrices. The matrices show the number of times each predicted ID (***x*-axis**) compares with the true ID (***y*-axis**). For example, for the success case (**left**), identity P3 was predicted 74 times, and in all 74 cases it was correct. In contrast, in the failure case (**right**), there were 66 cases where the method predicted identity T2 when the true identity was P1. Failure tends to occur when two people are wearing similar clothing, highlighting the reliance on appearance to distinguish and re-identify individuals. The images have been blurred and blotted to protect identities

Overall, our pipeline (schematized in Fig. 2) proved successful in (re)identification when videos contained 2–5 individuals and despite their being occluded, their exiting and re-entering the frame, and their moving past one another. To generate the results for the re-identification approach, we applied our pipeline to all frames in these videos, and superimposed the results on the original frames and exported the videos. We then uniformly randomly sampled ~7,000 frames from these videos and assessed with a human annotator whether the assigned identities were correct. Figure 3 presents the results of the re-identification. The solid red lines indicate the median person identification accuracy (i.e., 97.6% for 1,750 frames and *N* = 2, 91.9% for 1,207 frames and *N* = 3, 93.1% for 1,540 frames and *N* = 4, and 76.4% for 1,893 frames and *N* = 5). Table 2 provides a further breakdown of these results and highlights the total number of frames across all videos that were evaluated for correctness (>7,000 frames) as well as the average accuracy for any given number of unique individuals. The individual values reported in Fig. 3 (blue points) in the main text highlight that the downward bias originates from some instances of particularly challenging videos. We highlight a success and a failure case in Fig. 4, which includes confusion matrices for the individuals being re-identified. In the failure case, high similarity between the clothing of the child and one of the therapists resulted in regular misclassification.

**Table 2:** Results for re-identification across 36 videos varying in their number of unique individuals

Number of unique individuals	No. of videos	Cases	Cases (Excl. empty)	No. Correct	No. incorrect	Median accuracy (%)
*N* = 2	12	2,048	1,750	1,589	161	97.6
*N* = 3	8	1,423	1,207	1,009	198	91.9
*N* = 4	8	1,652	1,540	1,411	129	93.1
*N* = 5	8	1,948	1,893	1,191	702	76.4
Total	36	7071	6390	5,200	1,190	89.2

### Accurate movement tracking and pose estimation

**Fig. 5:**
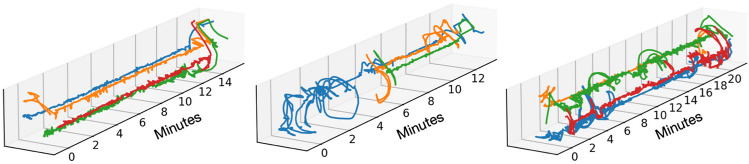
Three group session examples of *x-y* pixel coordinate positions/trajectories of individuals tracked throughout the course of the session are shown. Each individual is denoted by a different colour. These are generated by randomly sampling results from the re-identification process and manually verifying the identities

**Fig. 6:**
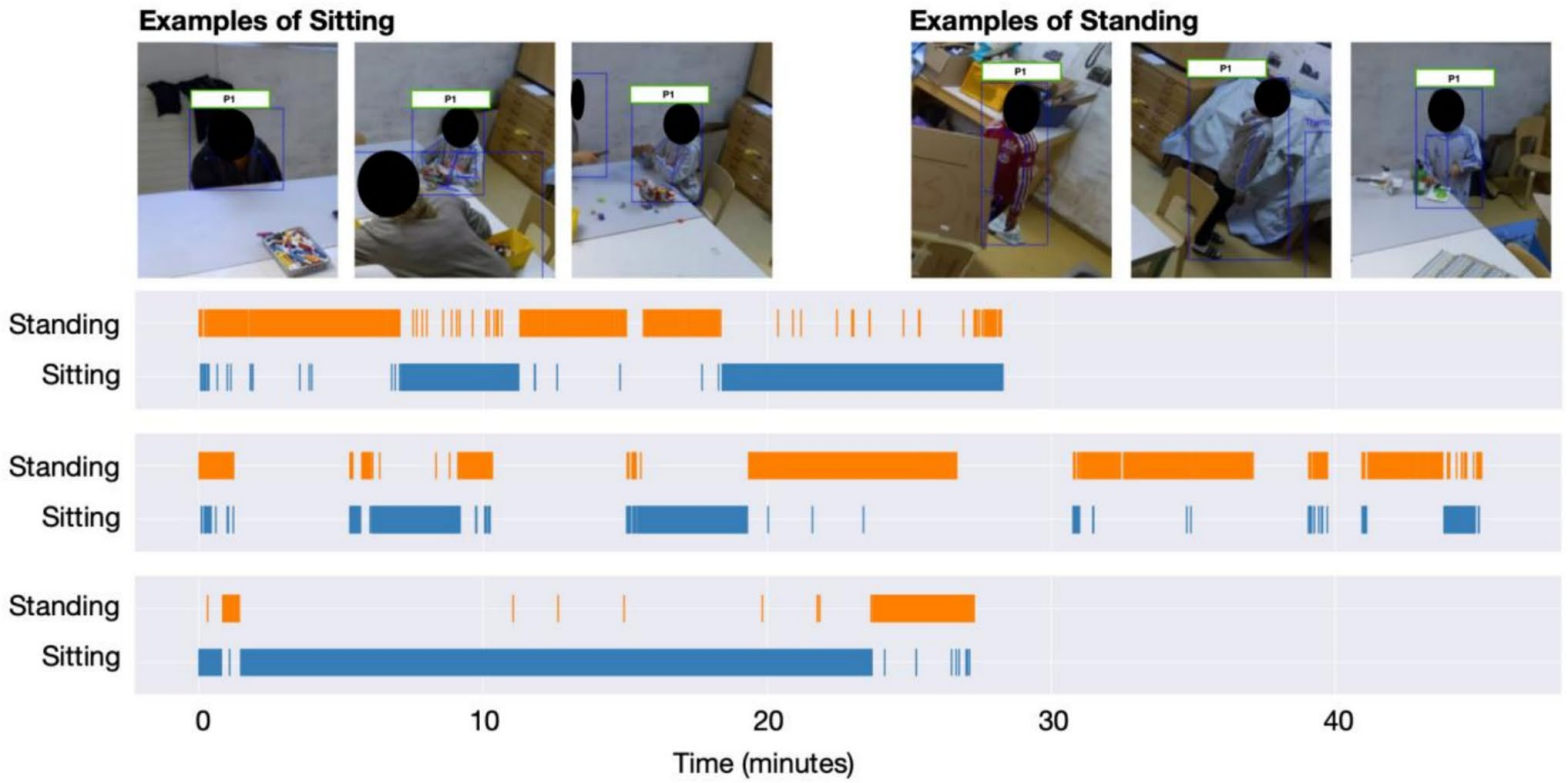
Upper left three: Examples of frames where the individual is sitting. Upper right three: Examples of where the individual is standing. These frames highlight the complexity of the scenes and the possibility for occlusion that our pipeline nonetheless surmounts. The bottom three horizontal plots display examples of event plots for when an individual is classified as sitting or standing throughout the course of a video

Position and pose were also successfully tracked by automatically annotating the skeleton configuration. On the one hand, we discerned whether (and where) each individual was moving in the space, and on the other hand, we classified their pose as sitting or standing. Figure 5 plots the *x-y* pixel coordinate position of the centroid of the person-detection bounding box over time for each individual from three different exemplar sessions. Alongside the re-identification, we also estimated the positions of the skeletons using OpenPose 2.0 (Cao et al., 2018). We used these skeletons to predict a range of poses, including whether each individual was sitting or standing. For this, we used a set of manually annotated ‘sitting’ and ‘standing’ labels applied to data from 10 sessions. The ground truth for these labels was generated from a human annotator using Elan (Lausberg & Sloetjes, 2009). We extracted ~237,000 frames from these videos and trained an XGBoost (Chen & Guestrin, 2016) classifier using a group-k-fold splitting process, where we trained on all frames and labels except for those from a hold-out, test video. The default hyperparameters (i.e., no tuning) were used. Specifically, we trained on skeletons from frames sampled from 9 out of 10 of the videos and made testing predictions on the tenth video. We then repeated the process until we obtained results for all possible train-hold-out video combinations (i.e., a group-k-fold cross-validation process). Across the dataset, sitting was predicted with precision = 0.78 ± 0.33, recall = 0.61 ± 0.32, and F1 = 0.63 ± 0.34 (mean ± std calculated across the 10 manually-annotated videos). Similarly, standing was predicted with precision = 0.67 ± 0.25, recall = 0.85 ± 0.23, and F1 = 0.72 ± 0.23. These metrics are weighted by the number of frames in each video, and F1 is the harmonic mean of the precision and recall. Overall, there were 135,000 frames for sitting and 102,000 frames for standing. Figure 6 provides some example frames where the individuals are either sitting or standing, highlighting the complexity of the scenes. The figure also provides three event plots for the pose of three example individuals, highlighting periods when they are either sitting or standing, as classified using our methods. These classifications can then be used, for example, to quantify time spent standing versus sitting, or to filter sessions according to child pose for purposes of downstream analyses.

### Accurate tracking of skeleton points

**Fig. 7:**
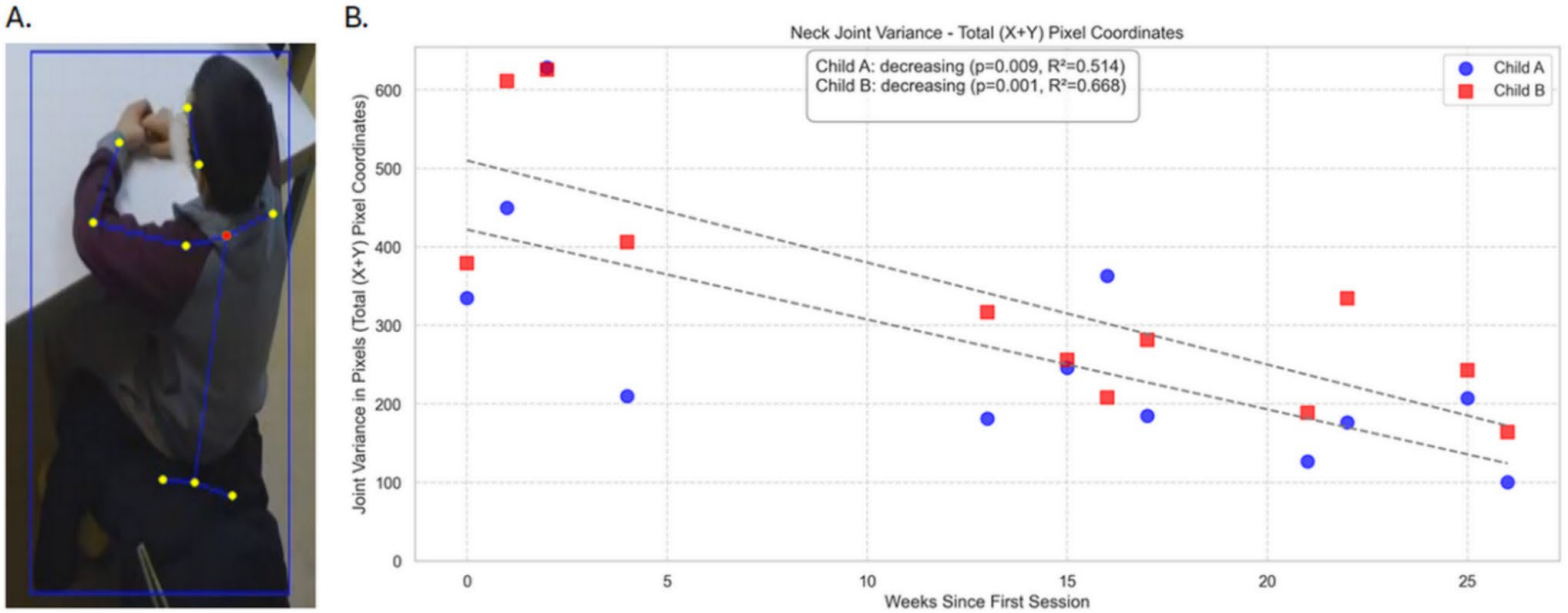
Panel **A** shows a single frame from an exemplar child and the identified skeleton points. In this analysis, we considered the neck point (red dot). Note that the face of the child has been blotted and blurred in the figure only. Panel **B** shows the neck joint variability summed across both X and Y coordinates for each recorded session for two children; the timespan of which was 26 weeks. In the case of both children, there was a significant reduction in this variability as a function of time (see inset)

Skeletons were also tracked both within a video and across videos for the same two individuals spanning 26 weeks. This allows for quantitative characterization of specific joints/points in the skeletons as an index of the child’s movement across different timescales and its variability. In Fig. 7 we show the variability in X and Y coordinates of the neck joint (red dot in Fig. 7a) and the analysis of changes in this variability across sequential video recording sessions. We demonstrate how tracking and frame-by-frame full-skeleton pose estimation, as implemented in the ADVANCE toolkit, can be used to estimate potentially clinically relevant indices from videos. Here, OpenPose captured the 25 anatomical key points, including the head, neck, shoulders, torso, hips, and limbs, for every frame in the video for two exemplar children. From these estimates, we applied the above-mentioned sitting/standing classifier to segment the recordings and extract only frames when the child was sitting, thereby ensuring that postural changes did not confound our analysis. This also demonstrates how intermediate pose categories can be leveraged to isolate relevant segments of behaviour within longer recordings. For each sitting frame, we measured the X and Y coordinates of the neck point and calculated their variance across the single video session, summing across both dimensions to obtain a total variance measure. Neck joint variance reflects the degree of head movement during sitting, with higher values indicating more frequent or larger movements, and lower values reflecting greater stillness. By plotting this variance across multiple video sessions for each child, we assessed whether and how physical movement during seated tasks changed over time. Both children in this example showed a statistically significant decrease in neck joint variance over the course of the 26 weeks of their sessions, suggesting reduced movement while seated. While exploratory, such a measure could serve as a clinically relevant marker of motor regulation and, by extension, the child’s engagement and/or attention, providing an objective complement to qualitative clinical observations.

## Discussion

The ADVANCE toolkit allows for the re-identification and pose-tracking of individuals in naturalistic contexts, overcoming limitations associated with alternative open-source methods. ADVANCE performs well despite cluttered environments, multiple individuals, occlusions, and movement variability. This is achieved through a combination of YOLOv8 (Varghese & Sambath, 2024), MediaPipe (Lugaresi et al., 2019), OSNet (Zhou & Xiang, 2019), and k-means clustering, while requiring no manual supervision. OpenPose (Cao et al., 2018) skeleton estimation and an XGBoost (Chen & Guestrin, 2016) classifier successfully labelled poses (sitting or standing). The ADVANCE toolkit operates in a naturalistic setting without requiring individuals to execute a set of predetermined tasks or to be in prescribed positions, while also interacting and moving in a space (here, a classroom) that has not been a priori configured to constrain or promote specific actions. With the ability to correctly identify individuals in such a setting, we anticipate expanding this work by analysing movement tracking around the space of the room, the number and type of interactions between individuals and objects or individuals with other individuals, and time spent on specific tasks.

Beyond their ethological validity, unrestricted scenarios cascade to reduce the current burdens on clinical infrastructure and staff. Recordings need not rely on clinicians’ presence or time for scoring, freeing them instead for treatment delivery and healthcare. Automated and quantitative annotation moreover yield metrics that even the trained human eye cannot easily quantify (e.g., velocity of movements, movement type, time and trajectory of interactions with objects or individuals, or gaze direction). Encapsulating these methods into a user friendly toolkit is an ongoing effort to render them widely accessible. Of similar importance is the fact that data such as videos of children or clinical populations cannot readily be uploaded onto cloud-based servers or public repositories, severely curtailing the palette of analytic tools available to researchers and clinicians working with such data. The ADVANCE toolkit thus provides a pipeline that can be operated locally in a secure manner conforming to institutional and regulatory constraints. More broadly, these methods could cross-fertilize parallel efforts in pose estimation and video annotation of behaviours, both in the laboratory and in the field.

Despite how essential rich behavioural information is to understanding and improving understanding of human behaviour and clinical treatments, Bulling et al. note that human observational coding, as a core tool for capturing such information, is prohibitively expensive in terms of its cost in time and training (Bulling et al., 2023). It requires that the human annotators are first trained to identify and record in a prescribed and consistent way, the phenomenon or phenomena of interest. Then, many annotation processes require the annotators to watch and annotate iteratively, second-by-second or frame-by-frame, various behaviours as they unfold in a recording. Furthermore, it is inherently challenging to ensure consistent and unbiased ratings between annotators, thereby making it difficult to guarantee interoperability, efficient collaboration, and unification of datasets from different research teams. These expenses have a concomitant negative impact on sample size and statistical power, requiring, as it does, an assignment of funds which could otherwise be used to recruit additional participants. Consequently, human observational coding does not readily scale to large datasets. These considerations seriously hinder progress in research involving analyses of video recordings, such as in the context of mental health research or educational sciences, thereby also affecting the design of effective interventions, training, and the dissemination and sharing of data. By developing the ADVANCE toolkit, we seek to shift this status quo for the broader scientific and clinical community.

An alternative that we had considered was to modify extant algorithms to improve their performance for scenarios like those we presented here. We ventured (without success) to adjust the hyperparameters for these algorithms to optimize them for long track lengths, thereby minimizing the chances that a previously identified individual would be assigned a novel identity following occlusion. Indeed, the results for StrongSORT presented here (which also uses YOLOv8 in its implementation to perform the initial person detection) follow from attempts to tune the algorithm and represent the best we were able to achieve with this alternative method. In our view, it was more productive to yield a relatively simple solution, which was specifically designed for our use-case and which performed well, than to undertake significant modifications to existing approaches which performed poorly ‘off-the-shelf’. We posit that our algorithm performs significantly better than these alternatives for two principal reasons. Firstly, there is no real-time requirement for our approach. As such, we are able to feed the frames of entire sessions into a clustering algorithm, thereby giving access to as many examples of representative identity information for that video as possible, before assigning identities to those clusters. Secondly, we were able to specify in advance the total number of unique identities within the course of the video. In contrast, StrongSORT (Du et al., 2023) has no such predefined number of identities, which is a strength on one hand, but one which cannot be leveraged for applications where the total number of unique identities is a priori fixed for each video.

Several other popular tracking algorithms exist, such as ByteTrack (Zhang et al., 2022) and DeepSORT (Wojke et al., 2017). However, we note that ByteTrack and DeepSORT have significant limitations as benchmark comparison alternatives. Firstly, ByteTrack does not have identification persistence or re-identification capabilities (i.e., if a person who was previously tracked becomes occluded for a sustained period, they are very unlikely to be successfully reassigned to the same track). Secondly, whilst DeepSORT was innovative for its time, its performance is notably lower than alternatives. StrongSORT therefore represents the closest current equivalent to our approach, in terms of both its recency and its implementation with YOLOv8.

It is worth considering the extent to which the ADVANCE toolkit could be applied to other scenarios where human behaviours are recorded. While not empirically tested here, the methods are themselves ‘agnostic’ with regard to the space itself. In fact, we deliberately chose a standard art classroom, reasoning that such a space is relatively cluttered with objects of varying sizes, shapes, and colours. We also deliberately allowed children and therapists to move freely within the space without asking them to modify natural movement or behaviour to suit recording constraints. In this manner, the ADVANCE toolkit should readily operate in settings that are more sterile, such as those in typical clinical assessments/recordings and more generally in other unconstrained, naturalistic, and cluttered settings. However, future research will be required to validate this conjecture empirically.

Future work could likewise capitalize upon the integration of multi-camera feeds to enhance pose estimation and tracking capabilities (Lee et al., 2022). Multi-camera setups offer the advantage of capturing individuals from multiple perspectives, which could mitigate challenges posed by occlusion or limited views from a single camera. However, this approach introduces several challenges, such as the need to synchronize data streams across cameras, the complexity of merging skeleton data from multiple sensors, and the computational overhead of processing multi-view data. Aside from these considerations, there are also logistic and practical aspects, such as costs of equipping rooms in such a manner as well as minimizing the impression of ‘being watched’ for participants (particularly paediatric populations). Nonetheless, such an approach could not only improve the robustness of tracking, but also enable analyses of a richer variety of behaviours and at a finer resolution, such as examining gaze direction in relation to specific objects or individuals (Erel et al., 2022, 2023), fine motor skills of the hand or other limbs, and other subtle gestures that are difficult for human annotators to capture. Continued innovations in this regard will assist with using video recordings to assess features of multimodal communication, including but not limited to gestures, multisensory speech signals, indices of joint attention, etc. More generally, these developments hold promise for further improving the ethological validity and utility of automated behavioural observation systems. Given the ubiquity of video recordings for observing behaviours and inter-individual interactions, the ADVANCE toolkit is poised to catalyse annotation and surmount many challenges of manual annotation.

## Data Availability

The video datasets analysed during the current study are not publicly available to protect each patient’s anonymity. Because of this limitation, we provide a sample video acquired in the same setting with only the adult art therapists that can be used to demonstrate the integrity of the methods introduced in this manuscript. This sample video is available at: https://osf.io/xrsnw/?view_only=9f09d98040d24616bf1c30af18ff31a3. Also included in this repository are the corresponding estimated skeleton poses and the ID mapping for each frame. The abovementioned analysis code is shared so as to allow readers to check the correctness of their implementation.

## Code availability

Code for the re-identification analyses is available at: https://osf.io/4mfsk/.
